# Correction: Canadian newspaper coverage on harm reduction featuring bereaved mothers: A mixed methods analysis

**DOI:** 10.1371/journal.pone.0297479

**Published:** 2024-01-18

**Authors:** The PLOS ONE Staff

## Notice of Republication

This article was republished on December 28, 2023, to correct errors in the first author’s name and affiliation that were introduced during the typesetting process. The publisher apologizes for these errors. Please download this article again to view the correct version. The originally published, uncorrected article and the republished, corrected articles are provided here for reference.

## Supporting information

S1 File Originally published, uncorrected article. (PDF)

S2 File Republished, corrected article. (PDF)
