# Supplementary material for: Canadian newspaper coverage on harm reduction featuring bereaved mothers: A mixed methods analysis
Source: PLoS One. 2023 Nov 27;18(11):e0294608. doi: 10.1371/journal.pone.0294608 (PMC10681218; doi:10.1371/journal.pone.0294608)
Supplement: S1 Table — (PDF) [file pone.0294608.s001.pdf]

S1 Table. Newspapers with Highest Circulation in Each Province/Territory, Canadian Harm Reduction Policy Project (CHARPP)

British Columbia	The Vancouver Sun, The Province [Vancouver], Times Colonist [Victoria], Courier [Kelowna], The Kamloops Daily News
Alberta	Calgary Herald, The Calgary Sun, The Edmonton Journal, The Edmonton Sun, Lethbridge Herald, Red Deer Advocate
Saskatchewan	The Star Phoenix [Saskatoon], The Leader-Post [Regina], The Times-Herald [Moose Jaw], Prince Albert Daily Herald
Manitoba	Winnipeg Free Press, Winnipeg Sun, The Daily Graphic [Portage La Prairie]
Ontario	Toronto Star, The Toronto Sun, Ottawa Citizen, The Spectator [Hamilton], The London Free Press
Quebec	Le Journal de Montréal, La Presse [Montreal], Le Journal de Québec, The Gazette [Montreal], Le Soleil [Quebec City]
New Brunswick	Times Transcript [Moncton], New Brunswick Telegraph Journal [Saint John], The Daily Gleaner [Fredericton]
Nova Scotia	The Chronicle Herald [Halifax], Cape Breton Post [Sydney], The News [New Glasgow], The Daily News [Truro], Amherst Daily News

Prince Edward Island	The Guardian [Charlottetown], The Journal Pioneer [Summerside]
Newfoundland and Labrador	The Telegram [St. John’s], The Western Star [Corner Brook]
Nunavut	Nunavut News/North, Nunatsiaq News [online], CBC North [online]
Northwest Territories	NWT News/North, CBC North [online], Yellowknifer [online], Northern
Yukon	The Whitehorse Star
