# Supplementary material for: Canadian newspaper coverage on harm reduction featuring bereaved mothers: A mixed methods analysis
Source: PLoS One. 2023 Nov 27;18(11):e0294608. doi: 10.1371/journal.pone.0294608 (PMC10681218; doi:10.1371/journal.pone.0294608)
Supplement: S2 Table — (PDF) [file pone.0294608.s002.pdf]

S2 Table. Database-specific Controlled Vocabularies for Harm Reduction

Canadian Newsstand Complete

(all(“harm reduction”) OR all(“needle distribution*”) OR all(“needle exchange*”) OR all(“low-barrier methadone clinic”) OR all(“syringe recovery”) OR all (“needle recovery”) OR all(“clean rig*”) OR all(“Evzio”) OR all (“Prenoxad Injection”) OR all(“Narcanti”) OR all(“Narcotan”) OR all(“methadone program*”) OR all(“fix room*”) OR all(“safer inhalation facilit*”) OR all(“safe smoking room*”) OR all (“low threshold maintenance”) OR all(“low threshold opioid”) OR all (“low threshold methadone maintenance”) OR all(“safe injection site*”) OR all(“safer injection”) OR all (“low threshold methadone treatment”) OR all(“low threshold opiate substitution”) OR all(“low threshold methadone”) OR all(“street nurse*”) OR all(“testing kit*”) OR all(“drug purity kit*”) OR all(“safer inhalation kit*”) OR all(“pipe exchange”) OR all(“clean stem*”) OR all(“clean pipe*”) OR all(“shooting gallery”) OR all(“syringe exchange*”) OR all(“safer inhalation”) OR all(“naloxone”) OR all(“supervised injection”) OR all(“opioid substitution”) OR all(“opioid replacement therapy”) OR all(“buprenorphine”) OR all(“needle program*”) OR all (“syringe program*”) OR all(“sterile injection”) OR all (“sterile needle*”) OR all (“clean needle”) OR all(“low threshold”) OR all(“suboxone”) OR all(“injection site*”) OR all(“injection facility”) OR all(“street outreach”) OR all(“street clinic*”) OR all(“narcan”) OR all(“Insite”) OR all(“drug checking”) OR all(“pill testing”) OR all(“safe injection*”) OR all(“methadone”) OR all(“supervised consumption facilit*”) OR all(“drug consumption room*”) OR all(“safe consumption site*”) OR all (“heroin assisted treatment”) OR all(“North American Opiate Medication Initiative”) OR all(“heroin prescription*”) OR all(“street drug testing”) OR all(“crack kit*”) OR all (“anti-overdose kit*”) OR all(“injection kit”) OR all(“crack pipe*”) OR all(“crack-pipe mouthpiece*”) OR all(“ankors”) OR all(“DanceSafe”) OR all(“needle kit*”) OR all(“Safe drug consumption”) OR all(“drug monitoring”) OR all(“adulterant screening”) OR all(“pipe distribution*”)) AND Pub.Exact("Star - Phoenix" OR "Whitehorse Star" OR "The Province" OR "The Ottawa Citizen" OR "Toronto Star" OR "Calgary Herald" OR "Leader Post" OR "Montreal Gazette" OR "The Spectator" OR "The Vancouver Sun" OR "Times - Colonist" OR "Edmonton Journal" OR "Winnipeg Free Press" OR “The Brandon Sun” OR “Kamloops Daily News” OR “Prince George Citizen” OR “The Sherbrooke Record” OR)

The following filters were utilized:

Publication Title: Calgary Herald" OR "Edmonton Journal" OR "The Province" OR "Vancouver Sun" OR "Times Colonist" OR "Winnipeg Free Press" OR "Ottawa Citizen" OR "Spectator" OR "Toronto Star" OR "Montreal Gazette" OR "Star Phoenix" OR "Whitehorse Star" OR "Leader Post” OR “Kamloops Daily News” OR “Prince George Citizen” OR “The Brandon Sun” OR “The Sherbrooke Record”

Publication Date: January 1st 2000 to December 31st 2016

Source Type: Newspapers

Eureka

(all(“harm reduction”) OR (“needle distribution”) OR (needle exchange*) OR (syringe exchange*) OR (“syringe recovery”) OR (“needle recovery”) OR (“Prenoxad Injection”) OR (“Narcanti”) OR (“Narcotan”) OR (safer inhalation facilit*) Or (methadone program*) OR (“safe injection”) OR (“low threshold opioid”) OR (“low threshold methadone”) OR (“low-barrier methadone clinic”) OR (safe injection site*) OR (“low threshold opiate”) OR (“safer inhalation kit”) OR (“pipe exchange”) OR (clean pipe*) OR (“safe inhalation”) OR (“naloxone”) OR (“supervised injection”) OR (“opioid substitution”)

OR (“opioid replacement therapy”) OR (“buprenorphine”) OR (needle program*) OR (syringe program*) OR (sterile injection*) OR (sterile needle*) OR (clean needle*) OR (“suboxone”) OR (injection site*) OR (injection facilit*) OR (“street outreach”) OR (“narcan”) OR (“Insite”) OR (“drug checking”) OR (“methadone”) OR (supervised consumption facilit*) OR (safe consumption facilit*) OR (safe consumption site*) OR (“heroin assisted treatment”) OR (“North American Opiate Medication Initiative”) OR (“heroin prescription”) OR (“street drug testing”) OR (“crack pipe kit”) OR (“anti-overdose kit”) OR (“injection kit”) OR (“crack-pipe mouthpiece”) OR (“DanceSafe”) OR (“Safe drug consumption”) OR (“drug monitoring”) OR (crack pipe*) OR (“ankors”) OR (“needle kit”) OR (“pipe distribution”))

The following filters were utilized:

Publication Title: “Lethbridge Herald" OR "Red Deer Advocate" OR "Telegraph Journal (NB)" OR "The Fredericton Daily Gleaner" OR "The Moncton Times and Transcript” OR “The Telegram (St. John’s)” OR “ The Western Star” OR “CBC North” OR “NWT News North” OR “Yellowknifer” OR “Amherst News (NS)” OR “Cape Breton Post” OR “The Chronicle Herald (Halifax, NS)” OR “The News (New Glasgow)” OR “ Truro Daily News (NS)” OR “Nunavut News North” OR “The Guardian (Charlottetown)” OR “The Journal Pioneer (Summerside)” OR “Prince Albert Daily Herald” OR “Moose Jaw Times Herald” OR “Winnipeg Free Press”

Publication Date: January 1st 2000 to December 31st 2016

Source Type: Newspapers

Factiva

“harm reduction” OR “methadone program” OR safe injection site* OR “low-barrier methadone clinic” OR “needle distribution” OR “needle exchange” OR “syringe recovery” OR “needle recovery” OR “clean rig” OR “Prenoxad Injection” OR “evzio” OR “Narcanti” OR “Narcotan” OR “fix room” OR “safer inhalation facility” OR “safe smoking room” OR “low threshold opioid” OR “low threshold methadone maintenance” OR “low threshold opiate substitution” OR “low threshold methadone” OR “street nurse” OR “testing kit” OR “safer inhalation kit” OR “pipe exchange” OR “clean stem” OR “clean pipe” OR “shooting gallery” OR “syringe exchange” OR “safer inhalation” OR “naloxone” OR “supervised injection” OR “opioid substitution” OR “opioid replacement therapy” OR “buprenorphine” OR needle program* OR syringe program* OR “sterile injection” OR “sterile needle” OR all “clean needle” OR “low threshold” OR “suboxone” OR injection site* OR injection facilit* OR “street outreach” OR “street clinic” OR “narcan” OR “Insite” OR “drug checking” OR “safe injection” OR “methadone” OR “supervised consumption facility” OR “safe consumption site*” OR “heroin assisted treatment” OR “North American Opiate Medication Initiative” OR “heroin prescription” OR “street drug testing” OR “crack kit” OR “anti-overdose kit” OR injection kit* OR “crack pipe*” OR “crack-pipe mouthpiece” OR “ankors” OR “DanceSafe” OR “needle kit*” OR “Safe drug consumption” OR “drug monitoring” OR “pipe distribution”

The following filters were utilized:

Publication Title: “Calgary Sun” OR “Edmonton Sun” OR “Winnipeg Sun” OR “Nunatsiaq News” OR “The London Free Press” OR “Toronto Sun” OR “Portage Daily Graphic”

Publication Date: January 1st 2011 to December 31st 2016
Source Type: Newspapers
