# Supplementary material for: Correction: Canadian newspaper coverage on harm reduction featuring bereaved mothers: A mixed methods analysis
Source: PLoS One. 2024 Jan 18;19(1):e0297479. doi: 10.1371/journal.pone.0297479 (PMC10796018; doi:10.1371/journal.pone.0297479)
Supplement: S1 File — (PDF) [file pone.0297479.s001.pdf]

PLOS ONE

RESEARCH ARTICLE

Canadian newspaper coverage on harm reduction featuring bereaved mothers: A mixed methods analysis

Heather Morris R. N.[1,2☯*], **T. Cameron Wild**[3☯], **Marina Giovannoni**[4☯], **Rebecca Haines-Saah**[5‡], **Jakob Koziel**[6‡], **Petra Schulz**[2‡], **Hauwa Bwala**[3‡], **Diane Kunyk**[1‡], **Tania Bubela**[7‡], **Elaine Hyshka**[3☯]

1 Faculty of Nursing, Edmonton Clinic Health Academy (ECHA), University of Alberta, Edmonton, AB, Canada, **2** Moms Stop the Harm, RPO Broadmead, Victoria, BC, Canada, **3** School of Public Health, Edmonton Clinic Health Academy, University of Alberta, Edmonton, Alberta, Canada, **4** Cumming School of Medicine, University of Calgary, Calgary, Alberta, Canada, **5** Department of Family Medicine, Faculty of Medicine and Dentistry, University of Alberta, Edmonton, AB, Canada, **6** Bissell Centre, Edmonton, AB, Canada, **7** Faculty of Health Sciences, Simon Fraser University, Burnaby, BC, Canada

☯ These authors contributed equally to this work.
‡ RHS, JK, PS, HB, DK and TB also contributed equally to this work.
* hmmorris@ualberta.ca

Abstract

A growing body of evidence suggests that news media which includes a sympathetic portrayal of a mother bereaved by substance use can increase public support for harm reduction initiatives. However, the extent to which such news media coverage occurs in Canada is unknown, and research has not documented how the news media in Canada covers such stories. We undertook a mixed-method secondary analyses of 5681 Canadian newspaper articles on harm reduction (2000–2016). Quantitative analyses described the volume and content of harm reduction reporting featuring a mother whose child's death was related to substance use while qualitative thematic analysis provided in-depth descriptions of the discourses underlying such news reporting. Newspaper articles featuring a mother whose child's death was related to substance use were rarely published ($n = 63$; 1.1% of total harm reduction media coverage during the study period). Deductive content analysis of these 63 texts revealed that coverage of naloxone distribution (42.9%) and supervised drug consumption services (28.6%) were prioritized over other harm reduction services. Although harm reduction (services or policies) were advocated by the mother in most (77.8%) of these 63 texts, inductive thematic analysis of a subset ($n = 52$) of those articles revealed that mothers' advocacy was diminished by newspaper reporting that emphasized their experiences of grief, prioritized individual biographies over structural factors contributing to substance use harms, and created rhetorical divisions between different groups of people who use drugs (PWUD). Bereaved mothers' advocacy in support of harm reduction programs and services may be minimized in the process of reporting their stories for newspaper readers. Finding ways to report bereaved mothers' stories in ways that are inclusive of all PWUD while highlighting the role of broad, structural determinants of substance use has the potential to shift public opinion and government support in favour of these life-saving services.

OPEN ACCESS

Citation: Morris R. N. H, Wild TC, Giovannoni M, Haines-Saah R, Koziel J, Schulz P, et al. (2023) Canadian newspaper coverage on harm reduction featuring bereaved mothers: A mixed methods analysis. PLoS ONE 18(11): e0294608. https://doi.org/10.1371/journal.pone.0294608

Editor: Juan A. López-Rodríguez, Rey Juan Carlos University: Universidad Rey Juan Carlos, SPAIN

Received: April 24, 2023

Accepted: November 3, 2023

Published: November 27, 2023

Copyright: © 2023 Morris R. N. et al. This is an open access article distributed under the terms of the Creative Commons Attribution License, which permits unrestricted use, distribution, and reproduction in any medium, provided the original author and source are credited.

Data Availability Statement: Please note that we have now uploaded the data set used for this study within the following data repository: https://borealisdata.ca/dataset.xhtml?persistentId=doi:10.5683/SP3/BNAZUY.

Funding: Funding: This work was supported by the Canadian Institutes of Health Research under grant CIHR; MOP137073; https://cihr-irsc.gc.ca/e/193.html) toTCW and EH; an infrastructure grant from CIHR to the Canadian ResearchInitiative in Substance Misuse (CRISM), Prairie Node under

grant # CRISMN139151(https://crism.ca/) toTCW and EH; Social Sciences and Humanities Research Council (SSHRC) DoctoralAward under grant # 752-2019-2510 to HM(https://www.sshrc-crsh.gc.ca/home-accueil-eng.aspx); Izaak Walton KillamMemorial Scholarship to HM (https://killamlaureates.ca/); the StolleryChildren's Hospital Foundation and the Alberta Women's Health Foundation undergrant #2221 to HM (https://www.wchri.org/); the Canada Research Chairsprogram through a Tier 2 Canada Research Chair in Health Systems Innovationunder grant # CRC TIER2 233345 which supports EH (https://www.chairs-chaires.gc.ca/home-accueil-eng.aspx). The funders had norole in study design, data collection and analysis, decision to publish, orpreparation of the manuscript.

Competing interests: The authors have declared that no competing interests exist.

Introduction

The drug poisoning crisis continues to surge across North America as over 100,000 overdose deaths have occurred in the United States in the 12-month period ending November 2022 [1] and over 34,000 apparent opioid toxicity deaths in Canada have been documented since January 2016 [2]. Parents bereaved by substance use have joined other key stakeholders in calling on provinces and the federal government to implement harm reduction measures in an effort to address the overdose crisis [3]. Rooted in compassion, human rights, and social justice, harm reduction is both a philosophy and an approach to the development of policies, services and strategies with an aim to help people who use drugs (PWUD) live healthier and safer lives [4]. Harm reduction supports, developed by and for people who use drugs, help to minimize many negative impacts of drug use, laws and policies without requiring that people stop using drugs [5]. Despite extensive research documenting the benefits of harm reduction services [6–9] expansion of these services in Canada remains politically contentious. As a result, harm reduction advocates–including mothers bereaved by substance use—have taken to using the news media to advance their advocacy messages in support of drug policy reform.

Harm reduction and the overdose crisis have been covered extensively by the Canadian news media in recent years. A 2018 Canadian population survey reported that the majority of Canadians (57.3%) had seen or heard media coverage related to harm reduction and 58.3% reported that they had seen or heard media coverage featuring a mother whose child had died from a fatal drug overdose [10]. While media reach is an important component of any media advocacy efforts, an essential component of any news media reporting involves 'framing', which emphasizes how an issue/problem is defined, what factors contribute to a particular issue/problem, morally judging the causes and potential effects of an issue/problem as well as outlining possible solutions to a problem [11]. Lancaster et al. [12] have reported on the role of the media related to drug policy reform and describes four different mechanisms by which media coverage frames messages or influences audiences. First, the media plays a key role in setting agendas by defining what issues are most salient, garnering the attention of members of the public and shaping public opinion. Second, the media frames an issue by showing the public how to conceive of a particular issue (e.g. opioid use disorder as a criminal problem vs. a public health problem). Third, the media can influence and change attitudes towards certain issues and fourth, the media has the potential to influence the thoughts and actions of politicians and policymakers. A recent analysis by Wild et al. [10] found in testing a social exposure model of public support for harm reduction that "media exposure exhibited a small, though statistically significant indirect effect on public support for harm reduction via stigma" (p. 13) suggesting that the media may play an important role in de-stigmatizing the public's view of people who use drugs.

Research on the framing of substance use in the news media has found that media coverage emphasizes both the criminality of drug use and individual choice/personal weakness [13–16]. For example, in their examination of over 1800 news articles between May 16 2013-May 16 2014, Kennedy & Valleriani [14] reported that news articles of Toronto Mayor Rob Ford's use of crack cocaine was permeated with criminal framing pertaining to gangs and drugs. However, the media-represented perspectives on people who use drugs and substance use may be changing. In contrast, a recent study by Wild et al. [17] suggests that the majority of Canadian media articles focused specifically on harm reduction adopted a health frame. Similarly, McGinty et al.[18] recently found that news media coverage of solutions to the overdose crisis in the U.S. has shifted from a criminal justice frame to one focused more on treatment, harm reduction and prevention. Media studies related to people who use illegal drugs have also explored stigmatizing language and treatment. While Quan et al. [19] found stigmatizing

language in a national Canadian newspaper decreased over time between 2009–2018, McGinty et al. [20] found 49% of the U.S. news stories they sampled randomly on the opioid epidemic contained stigmatizing terms with the proportion of such terms increasing over a 10-year period since July 2008. This is not inconsequential, as studies have found that the language and labels ascribed to substance use and services can impact beliefs around personal culpability [21] and support for harm reduction programming [22]. However, none of these studies focused specifically on news media featuring a mother whose child's death was related to substance use, a form of newspaper reporting which has become increasingly common and which has the potential to impact how substance use is framed in news media reporting.

In addition to members of the news media framing stories of substance use and the overdose crisis, framing is also formed by the subject/interviewee themselves. For example, as social movement actors, family advocacy organizations have utilized a social injustice frame [23, 24] in their efforts to redefine and transform current understandings of substance use while simultaneously demanding changes in policy, practice and government response to the overdose crisis. Social injustice has been defined as an issue ". . . that encompasses various forms of discrimination, inequality, and oppression rooted in social, economic, and political structures" [25, p.1]; thus some examples of social injustice frames utilized by bereaved mothers center on current efforts to decriminalize the personal possession of substance use in Canada as well as ensuring access to a safe supply of pharmaceutical-grade substances [3]. The use of 'authentic' voices in media reporting [26–28], while considered essential for gaining public support of controversial topics like harm reduction, may also run the risk of exploiting or sensationalizing individuals [27, 29]. Our own qualitative (non-mixed-methods) research highlighting the experience of family members' engagement with the news media found that while the news media were seen as powerful allies in advocating for drug policy reform, media advocacy also came at a personal cost to bereaved mothers whose stories were at risk of being misrepresented or sensationalized [30]. Participants in this study also shared concerns that journalists could be complacent or insensitive when interacting with bereaved mothers [30].

Emerging evidence indicates that messaging which includes a story of a bereaved mother can positively impact public support for harm reduction [31, 32]. However, the extent to which bereaved mothers have appeared in media coverage around harm reduction is presently unknown. As well, research to date has not explored how the media reports on bereaved mothers as authentic voices who have lost a child to substance use and harm reduction. To address these knowledge gaps, the objectives of the present study were to describe the volume and content of such newspaper media coverage, and to describe the underlying discourses seen in these newspaper texts. Our research questions were: 1) How often are bereaved mothers who have lost a child to substance use represented in newspaper coverage of harm reduction? 2) What are the underlying discourses seen in Canadian newspaper coverage on harm reduction which feature a bereaved mother whose child's death is related to substance use?

Methods

We adopted a mixed method study design to deductively and inductively analyze Canadian newspaper reporting on harm reduction which featured a mother whose child's death was related to substance use [33]. Our quantitative content analysis examined the extent to which *a priori* content of interest appeared in news texts using descriptive statistics [34] and our qualitative methods allowed us to ". . . consider the role of language and discourse in creating meaning and salience" [35, p.301]. Ethical review was not required as data analyzed was newspaper texts which were already in the public domain.

Source documents

Media coverage for this study was drawn from a larger corpus of newspaper articles that reported on harm reduction (2000–2016) retrieved as part of the Canadian Harm Reduction Policy Project (CHARPP) [17]. Additional details on the CHARPP program as well as a description of how the original corpus was systematically obtained from online sources across 10 provinces and 3 territories is described by Wild et al [17]. All original articles were retrieved from the following three databases: Canadian Newsstand Complete (Canadian News Stream), Eureka and Factiva. A total of 54 newspapers were identified by CHARPP research staff as having the highest circulation counts in each province and territory through the Canadian Newspaper Association's website (S1 Table). Online or other sources of print media were used to capture cases where regular newspaper circulation counts were not available (Northwest Territories and Nunavut). A list of search terms that equated with the term 'harm reduction' (as it pertained to illegal drugs) was developed with the assistance of a research librarian and validated by CHARPP co-investigators (many of whom are Canadian experts in the field of harm reduction) (S2 Table). Articles could include news reports, columns, editorials, op-eds or letters. Further inclusion/exclusion criteria are described by Wild et al. [17]. A total of 42,720 English articles were initially retrieved from 54 newspapers for 2000–2016 and following a rigorous screening process by two CHARPP research staff which included regular interrater reliability checks on randomly-selected batches of articles (yielding high agreement on all indicators), 5,681 newspaper texts were included in the final corpus. Each of these texts were coded by CHARPP staff for type of article, tone towards harm reduction, topic and type of harm reduction intervention. Interrater reliability was also high (exceeding 0.9 on 5 indices) amongst four research assistants when coding for tone, topic and interventions [17].

Article screening, inclusion criteria, and verification

A multi-phase screening procedure was used to identify a subset of harm reduction newspaper texts that featured bereaved mothers who lost a child to substance use from the 5,681 source documents (see Fig 1). First, with the assistance of an IT consultant experienced in conducting media analyses, a computer-based, algorithmic search was performed to identify texts from the corpus that contained any of the following words: 'mother, mom, parent, mum'. A total of 765 articles published between 2000–2016 matching these terms were initially retrieved. Upon reviewing these articles, we discovered that some articles featuring a bereaved mother were not included if their child was mentioned in the article without reference to their relationship with the bereaved mother. Thus, we re-ran our search by adding two additional search terms (son and daughter) which yielded a total of 3064 articles.

In a second screening phase, the 3064 potentially relevant texts were then screened by Rater A (HM) to see if they met the study inclusion criteria and to remove duplicates. Articles were eligible for inclusion in this study if they (1) described a mother whose child's death was related to substance use (e.g., overdose/poisoning, substance-related suicide, death due to an illness that was left untreated due to substance use, infection/communicable diseases acquired through substance use or substance-related vehicle fatality) and (2) if the mother was quoted in the article and/or offered some information or opinion in the article herself (articles presenting pseudonyms or anonymization for mothers were excluded). Following the initial article selection by Rater A (HM), 20% of the articles were randomly selected and evaluated for inclusion by Rater B (MG) to assess inter-judge reliability. This was initially undertaken in batches of 50 then followed by groups of 100, yielding a Cohen's Kappa coefficient of 0.94 across all batches. Disagreement was resolved by discussion/consensus with 3 articles being reviewed and decided upon by both coders. These screening procedures initially identified 66

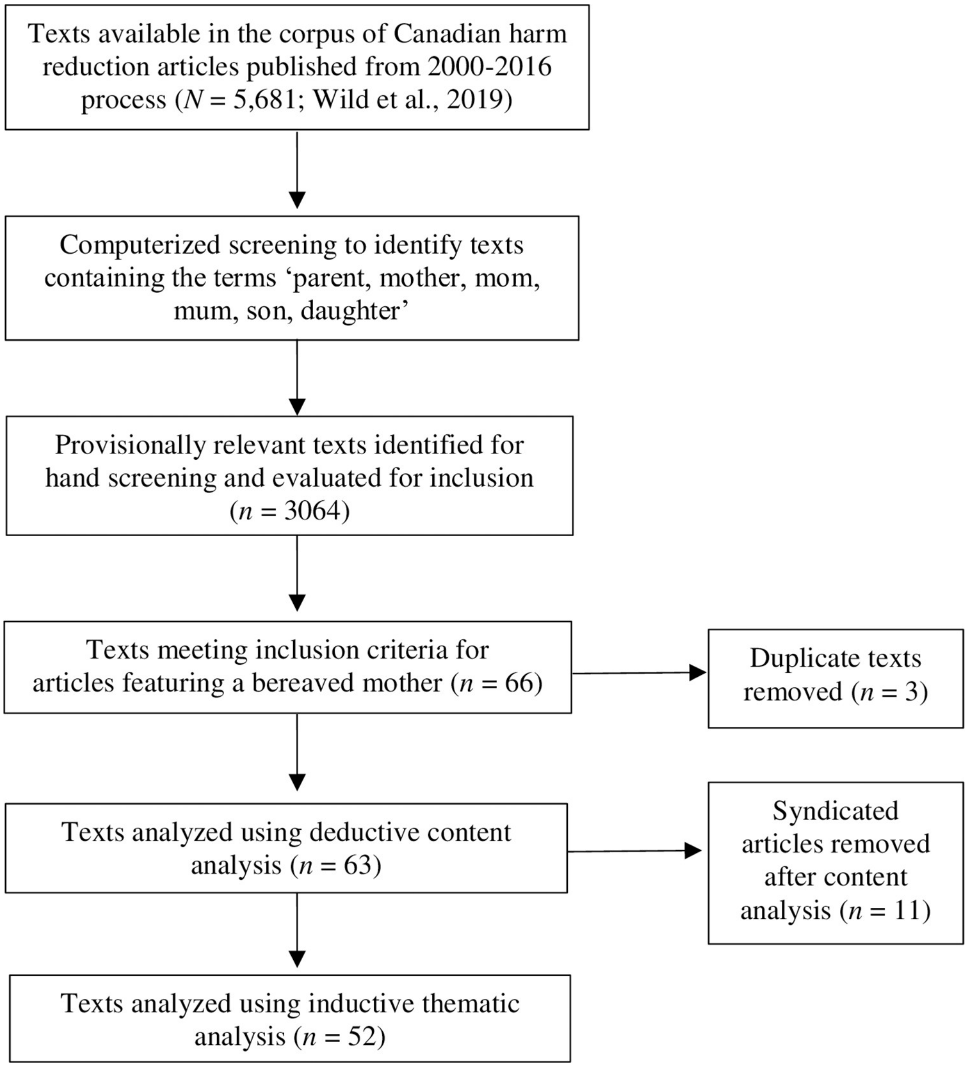

Fig 1. Screening and verification process. Screening and verification of texts first identified by Wild et al. (2019) which were subsequently evaluated for inclusion for both the content analysis and thematic analysis.

https://doi.org/10.1371/journal.pone.0294608.g001

newspaper texts for subsequent quantitative content analysis. Three texts which were exact duplicates from the same newspaper were omitted to yield a final population of 63 articles utilized for the quantitative content analysis. In a final screening step, we reviewed each of these 63 articles for their suitability for qualitative thematic analysis. This process revealed that 11 of the 63 included articles were syndicated texts appearing in multiple newspapers. Syndicated articles were retained for the content analysis as it allowed us to quantitatively capture and extract all content which appeared in different articles from different newspapers, but were removed for the qualitative analysis (by choosing the longer of the two versions when necessary), leaving a final set of 52 articles available for qualitative analysis.

Quantitative content analysis

The 63 news media texts identified via the screening procedures were then subjected to a quantitative content analysis [34, 36] to describe both the volume and content of Canadian harm reduction newspaper coverage (2000–2016) featuring a mother whose child's death was related

to substance use. Each included text was evaluated on 58 *a priori* codes grouped into 15 attribute domains. Four attributes were available from the initial analysis of the texts as described in Wild et al. [17]: *document characteristics*, *tone*, *dominant frame* and *interventions*. Document characteristics included title, source database, newspaper type, year of publication, and province of publication. Tone was coded as positive if the author was supportive of harm reduction; negative if opposed to harm reduction; and neutral if no opinion was expressed or if the article provided a balance of negative and positive views on harm reduction. The dominant frame attribute included 5 codes: criminal (discussion of illegal drug use and/or trafficking as it relates to harm reduction services); social welfare (impact of harm reduction services on communities, community perceptions of harm reduction interventions, and other social issues including moral/ethical dilemmas); political (government involvement or views expressed by government actors towards harm reduction); health (health outcomes of harm reduction and/or health care for PWUD in the context of harm reduction); or multiple perspectives on harm reduction where two or more dominant frames were present. Interventions were evaluated in relation to 9 codes: harm reduction (general principles or conceptual discussion of harm reduction), needle/syringe distribution, naloxone distribution, supervised injection/drug consumption, opioid agonist treatment, street-level outreach, safer inhalation kits, or drug checking. Research staff coded for the dominant intervention (that which was discussed most often) but had the option of coding multiple interventions as well.

In addition to the four attributes just described, each of the 63 articles were evaluated with regard to 11 attributes developed for this study, all of which were clearly defined using terms that were both mutually exclusive and exhaustive (see Table 1). Attributes captured in Table 1 were developed from the supporting literature, discussion amongst team members, findings from our own previous research [30] as well as discussion with one of the study authors (a founding member of Moms Stop the Harm) who had expressed an interest in many of these additional codes. Collecting these additional attributes also allowed us to more closely examine the prognostic and diagnostic framing undertaken by bereaved mothers [23]. Face validity and content validity of each of the variables [34] were initially assessed with the assistance of two co-authors (CW and EH) and our community advisor (PS) from Moms Stop the Harm. Following a training session and pilot testing of the coding form with 3 selected texts (which allowed for subsequent refinements), Rater A (HM) and Rater B (MG) independently coded the remaining 60 articles then met to review the data each extracted. Differences in opinion were discussed and a final decision on extracted quantitative data was made by consensus by the two reviewers. All quantitative data were recorded in Excel then imported into SPSS (27) to describe both the content and volume of the data using valid N and percentages for all items at the national level.

Qualitative thematic analysis

Following the quantitative analysis, we drew on Braun & Clarke [37] in undertaking a qualitative thematic analysis of 52 articles. While some qualitative purists might be more drawn to utilizing critical discourse or narrative analysis to analyze newspaper articles, we were drawn to thematic analysis for a number of reasons. First, as a method (e.g. narrative interviewing, observation, thematic analysis) and not a methodology (e.g. grounded theory, narrative, ethnography), thematic analysis can apply to a broad range of epistemological and theoretical perspectives [37]. Second, since thematic analysis focuses on analytical procedures related to coding the development of themes (most often inductively, but at times deductively), it is a method that can potentially be used to answer most research questions from different types of qualitative studies [38]. Finally, Braun & Clarke [37] were also one of the first to describe

Table 1. Characteristics of articles featuring a bereaved mother whose child's death was related to substance use (2000–2016).

Attributes Extracted Specific to Bereaved Mothers, Children and Families	Number (%) of newspaper articles
Is the child's history of drug use described in the	
newspaper story (yes/no)	
Yes	33 (52.4%)
No	30 (47.6%)
Is the impact of substance use on the family told in the	
newspaper story (yes/no)	
Yes	15 (23.8%)
No	48 (76.2%)
Is harm reduction advocated for (service or policy) by the	
bereaved mother (yes/no)	
Yes	49 (77.8%)
No	14 (22.2%)
If Yes, which one is named?	
Naloxone	17 (27.0%)
Supervised Consumption/Supervised Injection Service	14 (22.2%)
Good Samaritan Drug Overdose Act	1 (1.6%)
Harm Reduction Services in General	2 (3.2%)
Harm Reduction Services in Mental Health	1 (1.6%)
Safe or Regulated Raves	1 (1.6%)
Decriminalization and/or Legalization	5 (7.9%)
Drug Testing Kits	2 (3.2%)
Multiple	6 (9.5%)
Not Applicable (N/A)	14 (22.2%)
Is the bereaved mother advocating against harm	
reduction? (yes/no)	
Yes	1 (1.6%)
No	62 (98.4%)
Is stigmatizing language or terminology used in the	
article? (yes/no)*	
Yes	51 (81.0%)
No	12 (19.0%)
Is the perspective of someone who uses drugs (firsthand	
account) included? (yes/no)	
Yes	10 (15.9%)
No	53 (84.1%)
Is information on resources/support for people who use	
drugs (PWUD) or families provided? (yes/no)	
Yes	10 (15.9%)
No	53 (84.1%)
Does the bereaved mother call on a government actor to	
improve access to harm reduction or support harm	
reduction measures in some way? (yes/no)	
Yes	14 (22.2%)
No	49 (77.8%)
Is the name of an advocacy organization mentioned in the	
newspaper story?–Mother affiliated with or mentions	

(*Continued*)

Table 1. (Continued)

Attributes Extracted Specific to Bereaved Mothers, Children and Families	Number (%) of newspaper articles
this advocacy organization (yes/no)	
Yes	20 (31.7%)
No	43 (68.3%)
If so, which one is named?	
Moms United and Mandated to Saving Drug Users	
(mumsDU)	12 (19.0%)
**Moms Stop the Harm (MSTH)	2 (3.2%)
Ally Centre	1 (1.6%)
Jack's Voice	2 (3.2%)
Western Aboriginal Harm Reduction Society	1 (1.6%)
From Grief to Action	1 (1.6%)
Street Connections	1 (1.6%)
Not Applicable (N/A)	43 (68.3%)

*Stigmatizing language or terminology included the following terms which were selected from current journal and media style guides and current literature on person-first language [41, 42]: Addict, Alcoholic, Druggie, Drug Abuser, Drug User, Former Drug Addict, Junkie, Recreational Drug User, Substance Abuser, Substance User (or a version of such terms, e.g. injection drug user). Note that the names of advocacy organizations that contained these words were not coded as stigmatizing language.
**MSTH was created in 2016

https://doi.org/10.1371/journal.pone.0294608.t001

thematic analysis using a clear framework, making it one of the most widely utilized methods for qualitative data analysis today [39]. Our analytical process involved Rater A (HM) and Rater B (MG) independently co-coding 20% of the qualitative sample (purposively sampled for variation in length and style of article) to develop an initial list of nine broad codes: Portrayal of Child; Family and Substance Use Disorder; Description of Illegal Drugs and Use of Illegal Drugs; Mothers' Advocacy Messaging; Other Political Actors; Journalist Objectivity; Motherhood/Gender; Health Care System; and Business Interests/Capitalism/Neoliberalism. Both analysts met to discuss data coded to these broad categories and resolved any discrepancies by way of consensus. Utilizing NVIVO 12 to manage the data, Rater A (HM) read the remaining articles to classify text excerpts to these broad codes. This was followed by developing additional categories and subcategories within each of the broader codes. Data both 'within' and 'across' these categories and subcategories were compared and used to generate themes to characterize the discourses contained in these newspaper articles [37]. Methodological and analytical rigor were maintained via multiple strategies [40] including the systematic collection of articles, engaging in negative case analysis, regular consultations and co-coding amongst team members, memo taking, use of an audit trail, and reflexivity.

Results

Content analysis

A total of 63 newspaper texts which featured a bereaved mother whose child's death was related to substance use were identified from a larger corpus of 5681 harm reduction articles published between 2000–2016 [17], representing 1.1% of newspaper coverage of harm reduction during the study period. Of these 63 harm reduction texts, 92.1% were classified as news articles while 7.9% were opinion pieces. These articles were retrieved from all 10 provinces (no articles from the 3 territories) with Alberta (33.3%), Ontario (15.9%) and British Columbia

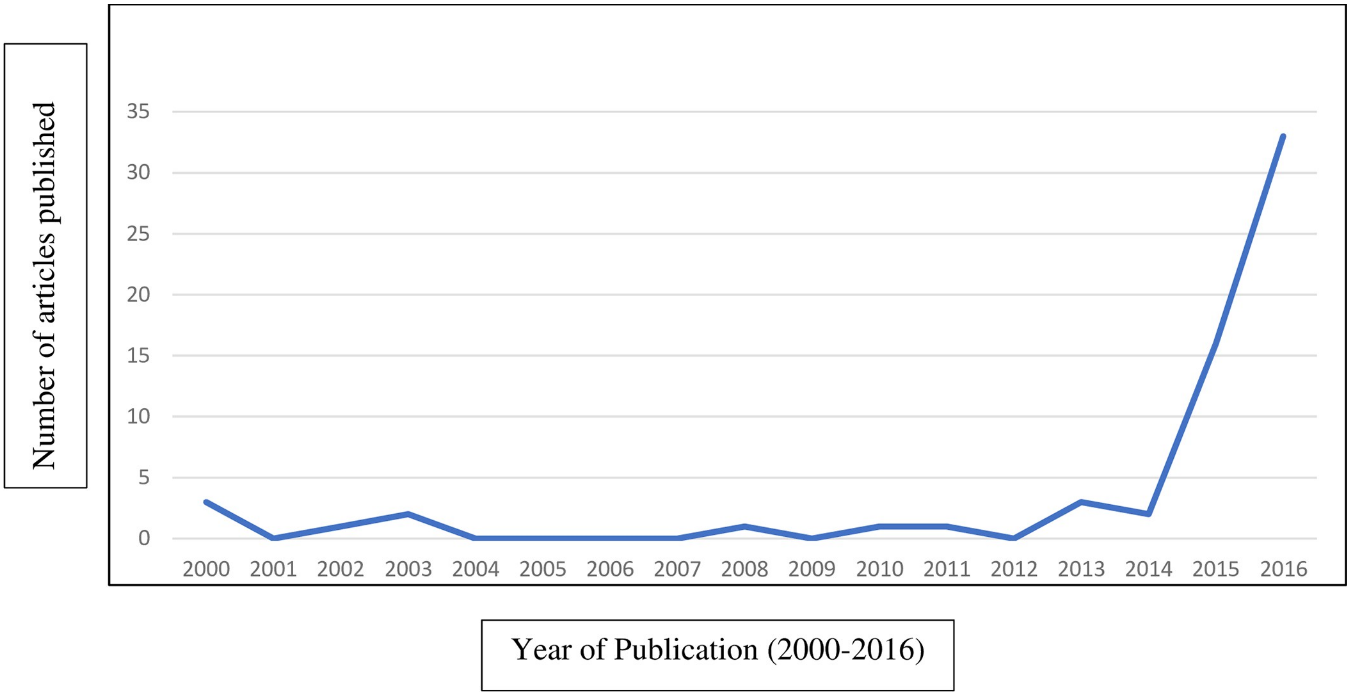

Fig 2. Volume of newspaper coverage. Volume of Canadian newspapers produced on harm reduction featuring a bereaved mother 2000–2016.

https://doi.org/10.1371/journal.pone.0294608.g002

(14.3%) having the largest proportion of texts. Fig 2 shows a sharp increase in the number of articles published from 2000–2014 (total of 14) in comparison to 2015 (16) and 2016 (33).

The dominant frame of the texts (as they related to harm reduction and not PWUD) was primarily focused on health perspectives on harm reduction (58.7%), followed by social welfare (25.4%), political (6.3%), multiple (9.5%) with criminal perspectives not appearing at all (0%). The overall tone towards harm reduction of these texts was split between neutral (50.8%) and positive (49.2%) with negative characterizations completely absent. Finally, coverage of harm reduction interventions primarily focused on naloxone distribution (42.9%), followed by supervised consumption services (28.6%), harm reduction in general (14.3%) and multiple harm reduction interventions (7.9%). Opioid agonist treatment (3.2%) and drug checking (3.2%) were seldom published and texts describing needle/syringe distribution, safer inhalation kits, and street level outreach were not published at all. Additional descriptive details of content characteristics specific to bereaved mothers and families over this 17-year period is shown in Table 1.

Thematic analysis

Three primary themes were revealed in the thematic analysis. First, notwithstanding the quantitative findings reported earlier that mothers' messages centering on the need for harm reduction initiatives were common, the media emphasized mother's expressions of grief and loss, thereby positioning their emotional pain as a key element of the news story. Second, we observed that articles tended to highlight individual over structural determinants of substance-related harm. Our third and final main observation was the rhetorical division created in articles, between different groups of people who use drugs.

The news media's emphasis on a mother's grief. One of the ways that news media told the story of bereaved mothers was through sharing their first- hand accounts of years spent supporting their child using substances, their grief, and subsequent advocacy for drug policy reform.

Mother's advocacy messaging centered on a wide variety of harm reduction initiatives, most often naloxone, supervised consumption services and ending the stigma towards PWUD and their families. Many shared personal anecdotes about their child while emphasizing that their child might still be alive had harm reduction services been available at the time of their death:

> *"'I honestly think if something like this [supervised consumption site] had been here, [Daughter] would have come here instead of being up the street in a hotel room with two girls who didn't know what to do when she overdosed.'. . . 'We used to talk about it and she never wanted to die,' said [Mother], who struggled not to cry as she talked on the sidewalk in front of the injection site, where camera crews, local residents and politicians mingled." (The Vancouver Sun, Sept. 16, 2003)*

Newspaper reporting on bereaved mothers' voices emphasized their experiences of loss and grief, including descriptions of mothers' emotional reactions, in addition to their advocacy messages. It was not uncommon to see a mother's personal experience used as a rhetorical device at the beginning or ending of an article to draw the reader in to the newspaper text or increase its resonance for the reader. At times the text would report on the moment when the mother was notified of her child's death; at other times, how mothers lived day-to-day with the grief, or how it informed their advocacy work:

> *"'Our family has had the hardest task ever—learning to live without [Son]', [Mother] said. But instead of hiding what happened to him, the family has chosen to speak out to reverse the stigma around drug users." (Edmonton Journal, Sept. 1 2015)*

> *"'There's a misconception that you heal after a death. You don't', said [Mother], [Mother's age]. 'You just learn how to integrate that loss. It's a daily struggle. I will never see my son again, I will never see him marry, I will never have grandchildren. It's the loss of all your hopes and dreams.'" (Toronto Star, July 29, 2013)*

Media texts were employed to showcase and elicit emotions of "every parent's nightmare" (The Ottawa Citizen, Sept. 11 2000) with a distinct refrain of how mothers ". . . fought back tears. . ." (The Winnipeg Sun, Sept. 1 2016), their ". . .voice cracked with emotion. . ." (The Winnipeg Sun, Oct. 21, 2016), how a mother ". . .struggled not to cry. . .." (The Vancouver Sun, Sept. 16, 2003), or spoke ". . . in a quavering voice. . ." (The Globe & Mail, May 10, 2008). Very few of the articles included in our sample included the perspective of a father alongside the perspectives shared by mothers and when this did occur, rarely was a father's emotions expressed as part of the newspaper text or advocacy messaging. Rather, mention was made of a father's career, their own relationship with their child, and the efforts they made to support their child struggling with substance use.

In nearly half of the included articles we observed a description of the circumstances around the time of the child's death, often revealing a level of detail that appeared to be excessive or which might be employed for the purpose of 'shock value.' Such descriptions centered on the location of the child's death, the fact that the child passed away alone or that the child lacked support of others who were present at the time of death. At other times, the story shifted to the moment when the mother or family were notified or became aware of their child's death. Thus, while bereaved mothers spoke courageously of the life and death of their child, they lay bare the possibility that in doing so, their child's death (and their emotions around it) might become the story itself. This discursive approach functions to focus the reader's attention on the harmful outcomes or collateral damage of substance use. Such was the case with a

text that appeared in the Calgary Herald where the location of the child's death was mentioned twice, both at the beginning of the text and again at the end: *"In September, [Son's] body was discovered in the janitor's closet of an underground parkade, dead from what his parents believe was a fentanyl overdose. He was 19. His body may have been there, all alone, for days"*. (Calgary Herald, Dec. 18, 2015). Such graphic and sensationalistic reporting appeared to be woven into longer, feature-length articles containing harmful stereotypes. Thus, while mothers shared their messages of advocacy, newspaper reporting emphasized their experiences with death and grief in an effort to provide the 'personal angle'—one that may be seen as necessary to captivate readers' attention and maintain interest in the news story [43].

Highlighting individual over structural determinants of substance-related harms. In describing the children who had passed away, nearly all of the articles described some biographical features of the child whose mother was featured in the news story. Most often this included a description of the child including their personal interests, aspects of their career or personality traits that endeared them to those who knew and loved them. This proved to be a critical feature of the analyzed texts, emphasizing the tremendous personal loss and reminding readers that those whose deaths were related to substance use are greatly loved, had lived a life of purpose and were worthy of remembrance. The representation of substance-related death in news stories featuring bereaved mothers would often reveal how children struggled with mental health or substance use from an early stage in life. This helped to reveal a wider personal context for the reader to elicit what individual factors in life may have played a role in the development of a substance use disorder (if present) and how. Longer articles that adopted a more narrative approach in reporting were more likely to discuss the negative impact that the child's substance use had on the child's life and the relationships with those around them. In doing so however, they also ran the risk of inflating the description of the child's struggles with substance use to a level that approached sensationalism. Such was the case in the following story featured in the Calgary Herald (Dec. 18, 2015):

> *"The young man, once worshipped by his younger sister and brother, began to slip away when he got into fentanyl. He spent six months on the street, later telling his mom, 'You don't even want to know what I did.' He stole family heirlooms. He pawned his mother's engagement ring. He withdrew $6000 from his mom's bank account by forging cheques. He also convinced his parents to pay off a $10,000 drug debt and he called them to bail him out of jail."*

Reporting such as this positions individual determinants of substance use over structural determinants of health which has been defined as "cultural norms, policies, institutions, and practices that define the distribution (or maldistribution) of . . ." [44, pg. 230] social determinants of health. Rarely were social/structural factors such as unstable or inadequate housing, unemployment, health care system failures or people's encounters with racism/colonialism brought forth in an effort to explain or provide context as to why some people struggled with substance use or experienced related harm. When poverty was discussed, it was most often in the context of underserved communities where people used drugs in 'back alleys' or 'grim hotels.' Very few texts spoke of the impact of substance use on Black or First Nations communities, and when this did occur, there was no mention of how racism or colonialism intersected with substance use or contributed to disproportionate rates of drug use or interactions with the criminal justice system. Two exceptions to this theme were noted, however. First, critiques of the health care system were reported as a common refrain shared by many mothers who emphasized how they had tried to help their child access and navigate community supports (e.g. psychological counselling, opioid agonist treatment, detox or residential services) often without success. Such was the case with [Mother] who was quoted in the Winnipeg Sun (Oct.

21, 2016) as stating, "'*The issue my son was faced with was there was a huge struggle over six years trying to get him help with the little resources we have.'[Mother] said private treatment is more widely available but only for those who can afford the price, which can be hundreds of dollars per day."* The other exception to the lack of discussion around structural forces pertained to a few articles which addressed drug prohibition and the need for decriminalization as a policy solution to the overdose crisis. An article in the Edmonton Sun (April 11, 2016) highlighted a bereaved mother who spoke of the need for society to address punitive drug laws:

> *"The report from the Johns Hopkins Bloomberg School of Public Health and the academic medical journal The Lancet called for the decriminalization of minor and non-violent drug use . . . and greater investments in health and social services for drug users. For [Mother], those changes are a no-brainer and she's hoping this time the UN and its member states will listen and react in kind. 'Our youth. . . our seniors are dying and things have to change. Our prisons are full of people with nonviolent drug crime and their lives are devastated with the stigma of having a criminal record and it hasn't worked. We have to do something differently. . ."*

Rhetorical divisions between different types of people who use drugs. Journalists took great efforts to describe a mother's child in terms that would be familiar to their readers. In doing so, this often created a rhetorical division between different groups of people who use drugs. One such way this occurred was in distinguishing between people who used drugs occasionally ('recreational users' or people 'experimenting') and those with a longer history of substance use. Another example was when journalists called upon the use of middle and upper income markers when describing the child and family in the story. This might be done subtly, such as when journalists mentioned a luxury car driven by someone in the story, a particular neighbourhood where the child grew up or the educational attainments or career of the child and/or his or her parents. In the few instances when a father was included in the story alongside mothers, his career was almost always mentioned. Such was the case with an Ottawa Citizen article which highlighted that the child's father was an accountant with a chemistry degree and that occupations of other immediate male family members included a pharmacist and a physician (The Ottawa Citizen, Sept. 11, 2000). Other articles explained that the father of a deceased child was a local news reporter (The Spectator, Oct. 1, 2016) while another text mentioned that the child was "the son of a power engineer and a stay-at-home mom,. . ." (The Calgary Herald, Dec. 18, 2015). At times however, a binary representation of what constituted a person who used drugs was created, contrasting the 'innocent' child who had passed away and other individuals who use drugs:

> *"Before he died, [Son] looked nothing like the self-destructive wreck that is the public's image of a heroin user. He was a bright 19-year-old, an excellent student who had recently graduated from high school. He was also an independent, creative spirit who read widely, including Henry Miller, James Joyce, Anais Nin and many others . . . Like many other bright teenagers, [Son] wanted to try drugs not to escape emotional pain, but in the romantic, perhaps foolish, hope of opening 'the doors of perception,' . . .. Contrary to popular wisdom, most people who use illegal drugs, even cocaine and heroin, do not go on to become regular users, let alone addicts. It is especially unlikely that a well-adjusted teenager experimenting with drugs out of intellectual curiosity will become addicted. Whether that teenager will get through this experimental phase unscathed is another matter." (The Ottawa Citizen. Sept. 11, 2000)*

This story depicts a stark comparison between a child who lived an apparently privileged and introspective existence as a bright 19 year-old who excelled at school and other people who use heroin as 'self-destructive wrecks.' In doing so, the journalist creates a wedge between low and higher income/status families impacted by substance use which ultimately contributes to further stigmatizing all people who use drugs while also obscuring the structural determinants of substance-related harm. One exception however, was an article which featured a first-person narrative of a bereaved mother who was herself someone who used illegal substances:

> *"[Mother] is hoping that [inhalation space at Insite] happens. Her 20-year-old daughter died in 2008 after overdosing on crack cocaine while smoking by herself. [Daughter] wasn't an injector, and though overdoses among smokers are rare, [Mother] said the case highlights the danger inhalers can face. 'I believe if there had been a safe inhalation site, she'd be alive today,' [Mother] said through tears. [Mother],[Mother's age] is president of the Western Aboriginal Harm Reduction Society . . . She said she smokes crack every day and would use an inhalation room if one were to open." (The Globe & Mail, May 14, 2011)*

Stigmatizing terms were used to describe the children of bereaved mothers who lost their lives. Such was the case with a Vancouver Sun (Nov. 7 2002) article entitled "Mom of Dead Addict Backs West-Side Injection Sites". Other terms used to describe the children in these stories included 'fentanyl-addicted kids', 'family members of addicts', or 'streetwise addicts'. The use of such terms, which serve to 'other' PWUD [45] was even more apparent, however, when speaking of people who used drugs in general (as opposed to the child who had passed away). Those who used drugs in general were often and repeatedly referred to as 'addicts', 'drug users', or 'self-admitted or confirmed drug users.' Language used to depict the use of drugs included words such as 'drug misuse' and terms used to describe the overdose crisis or harm reduction services itself could also be reduced to stigmatizing terminology. Examples of this included a Spectator (Oct. 1, 2016) article entitled 'Bad Medicine'; the Chronicle Herald (Oct. 15, 2016) article entitled, "Pop-up Drug Site; Addicts Get Help to Curb Overdoses"; a Globe & Mail (July 11, 2013) article entitled 'Board of Health is First to Endorse Drug Sites'; and The Edmonton Journal (Oct. 28, 2016) piece entitled "Alberta Eyes Drug-Use Havens; Fentanyl, Opioid Deaths Force Study of Controversial Clinics." One article featured in the Ottawa Citizen (Sept. 11, 2000) mentioned that ". . . the usual way public health officials spot bad drugs is to follow the corpses", further stating:

> *"Marginalization is key to understanding the predicament of drug addicts. In the public's mind, drug addicts are derelicts who live in squalid ghettoes. But drug addiction alone is highly unlikely to reduce people to that miserable state. It is the criminalization of what the addict needs, and the labelling of addicts as criminals, that turns them into human wreckage. The millions of prescription drug addicts worldwide do not live in slums, and relatively few alcoholics look like the walking dead of Vancouver's east side. But 'if you're dependent on something illegal, whether it's a drug or whatever,' says [Policymaker], 'that's the way you're going to look.'"*

While this author speaks to concepts such as marginalization and criminalization of drugs, their own language in this text further promotes stigma towards all PWUD. By distinguishing between 'prescription drug addicts' and 'alcoholics' and those described as 'the walking dead of Vancouver's East side', this author further contributes to polarizing different people who use different types of drugs.

Discussion

The objectives of this study were to describe: 1) the volume and content of Canadian newspaper coverage of harm reduction which featured a bereaved mother who has lost a child to substance use and, 2) describe the underlying discourses seen in such newspaper texts. Our screening of 5681 Canadian harm reduction news articles published from 2000–2016 yielded a total of 63 articles that featured a bereaved mother who had lost a child to substance use between 2000–2016, representing 1.1% of all harm reduction newspaper reporting during that time. Although only 1.1% of the harm reduction newspaper texts we examined included bereaved mothers, we suspect that the volume of such reporting would have been greater had we included articles dated after 2016 as well as those pertaining to the overdose crisis in general instead of those focused specifically on harm reduction. The volume of such articles increased over time between 2000–2016 with the most notable increases seen between 2014–2016. This mirrors similar findings from other studies that have tracked a sharp increase in media reporting on overdose during 2015–2017 relative to earlier years [15–19]. Increased coverage of bereaved mothers' media also aligned with the emergence of two pro-harm reduction Canadian family advocacy groups (Moms United and Mandated to Saving Drug Users or mumsDU and Moms Stop the Harm or MSTH) in 2015 and 2016 respectively. As the number of deaths due to a poisoned drug supply have escalated, members of these two national high-profile organizations have continued to cultivate strong relationships with the press since 2016, by issuing press releases, holding press conferences and/or inviting journalists to community events where their messages are shared. As a result, they have often been sought after by journalists to comment on a wide variety of policy solutions such as community distribution of naloxone and support for the passage of *the Good Samaritan Drug Overdose Act*, *Bill C-224* in Parliament. More recently, bereaved mothers have been asked to share their stories and comment on the expansion of supervised consumption services, decriminalization of drugs and the availability of a legal and regulated safe supply of opioids and other drugs across Canada [46]. Such activities are significant because ". . . journalists, who consistently deal with scarce resources, tight deadlines, and limited space for their stories, rely heavily on known and legitimate sources of information" [47,p.2]. In other words, those who belong to a larger, more formal advocacy organization with greater resources, expertise and a strong reputation with mainstream outlets are more likely to gain increased news coverage than individuals who are acting independently or with less formal support [48–51].

Our quantitative content analysis revealed that the dominant frame in news reporting on harm reduction that featured bereaved mothers focused on health perspectives of harm reduction (58.7%) with a criminal perspective frame completely absent amongst the texts reviewed. Evidence that harm reduction services or policies were advocated for by the bereaved mother in the majority of texts (77.8%) is important to note as it suggests that bereaved mothers' messages are being communicated to the public in their interactions with journalists. Combined with our findings that the overall tone of these texts was either neutral or positive (and not negative) towards harm reduction, these results suggest that harm reduction reporting including bereaved mothers have positioned harm reduction as in line with public health practice, defined here as ". . . an approach to maintaining and improving the health of populations that is based on the principles of social justice, attention to human rights and equity, evidence-informed policy and practice, and addressing the underlying determinants of health" [52, p. 4]. Dominant coverage of naloxone distribution versus other types of harm reduction interventions was not surprising given that Health Canada was in the midst of a widespread public consultation process on the non-prescription use of naloxone in 2016 [53]. As well, our

population of newspaper articles retrieved 2000–2016 predated the wider expansion of supervised consumption services beyond what had been offered in Vancouver since 2003.

We also observed that the perspective of people who currently use or used drugs was largely (84.1%) absent from these texts and as such, mothers 'became the voice' not only for families directly impacted by substance use but also for people who use drugs themselves in these texts. There are a number of possible explanations for this. As a study design issue, our inclusion criteria only specified that articles be included that featured a bereaved mother whose child's death was related to substance use. Other texts in the main corpus of newspaper articles pertaining to harm reduction most certainly includes PWUD and not mothers. Nonetheless, one might have expected that more of the texts we examined would had included the perspective of both bereaved mothers and PWUD together given that the thoughts and opinions of people currently using drugs would be an important perspective to include given their expertise in understanding drug use and that their lives are most directly impacted by harm reduction initiatives. At the time these articles were written, journalists may have questioned the relevance of including PWUD in these news stories or may have simply been drawn towards interviewing those who share similar characteristics with themselves or the readers who subscribe to their newspapers. Another possible reason however likely relates to the widespread stigma associated with the criminalization of drug use and lack of safety that many PWUD have in talking openly and honestly about their use of drugs. While there are some examples of high-profile professionals publicly disclosing current use of illegal drugs [54], the fear of personal and professional repercussions prevent many PWUD from publicly disclosing their current or past use of illegal drugs with members of the news media.

In-depth thematic analysis of these texts revealed that newspaper reporting emphasized the grief experienced by mothers. This maternal grief is central to the stories shared by mothers and those conveyed by the news media. Fine [55] argues that social movement actors are not only exemplified by shared beliefs and actions but are also represented by a shared set of narratives or stories. Such stories are instrumental ". . .internally to cement [social movement] members in shared understanding and externally to convince outsiders through example that the cause is just. Along with the mobilization of bodies, narratives are the greatest assets of any social movement to create change" [55, p.141]. Such narratives are also important from the perspective of journalists as they are used to draw in the reader and sustain interest in the news article. However, the sharing of these narratives can come at a cost to social movement organizations if it contributes to their stories being sensationalized and their advocacy messages being obscured. Snow [24] refers to this as a *framing hazard*, which has the potential to limit the *frame resonance* that a social movement may have with one's target audience. These findings complement prior research with bereaved mothers showing that those participating in media advocacy feared that their messages might be overshadowed by their stories of grief when speaking with the press [30].

Our thematic analysis also revealed that very few texts emphasized structural determinants of drug poisoning and substance-related harm, which aligns with the findings of other studies describing media reports of the overdose crisis and drug consumption rooms [16, 19, 35]. It is unknown whether journalists deliberately downplayed structural factors in their reporting or whether mothers themselves did not discuss them during the interview. Iyengar's seminal work on media framing [56] points to the role that thematic stories play in shaping how television viewers understand a problem. When news stories expand context by presenting background information and highlighting social and political forces in addition to showcasing people and events, viewers are more likely to acknowledge that governments and other institutions are responsible for attending to social and health problems rather than focusing on individual culpability. This finding has been supported in one drug policy communications study

whose authors found that, "depicting the barriers to treatment faced by a low SES woman [for opioid pain reliever addiction during pregnancy] lowered support for punitive policies and increased support for expanded insurance coverage for treatment" [57, p.873].

One reason why these wider structural factors were largely absent may relate to the fact that the mothers featured in these articles may not have been directly impacted by some of these factors themselves (e.g. racism and poverty). However, the sole article that we retrieved featuring an Indigenous mother who used substances herself made no mention of the disproportionate harms faced by Indigenous people [58–60] or the wider structural forces (colonialism, intergenerational trauma, impact of residential schools) that may have contributed directly or indirectly to her own family's vulnerabilities. A number of authors have been critical of media attention surrounding the North American overdose crisis stating that sympathetic narratives of substance use have highlighted individuals and families from upper income groups and those who identify as white rather than those representing Black and Indigenous communities [16, 61–65]. While we did not assess newspaper texts in relation to racial/ethnicity or social class attributes in our content analysis, we did observe a number of middle-upper income class markers (i.e. names of neighborhood, brand of car, education attained or occupational groups) woven throughout the texts suggesting that the articles were written for an audience of similar background. Such framing is in line with a narrative shared by bereaved mothers themselves highlighting that 'this could happen to anyone'. However, we also observed that journalists would at times write narratives which contrasted people who use drugs from more affluent communities with those who came from socially marginalized ones. Even without making a direct comparison to people who use drugs, highlighting details around socioeconomic status has the potential to 'other' those families and PWUD who are not part of upper-middle class white society. This is problematic on its own and should not be used as a rhetorical device to elicit sympathy or empathy for some but not all PWUD. Journalists, on the other hand, may believe that it is important to feature people who use drugs from more affluent communities in order to remind readers that not all people who use drugs come from marginalized groups, thereby contributing to de-stigmatizing efforts themselves. Others may believe that featuring people from more affluent communities may generate a more positive response from the general public towards harm reduction. Such an approach is in line with research conducted by Kennedy-Hendricks et al. [57] who found that the narratives that included a woman of high socioeconomic status (SES) who experienced opioid use disorder during her pregnancy were less likely to elicit perceptions of individual blame and decreased support for punitive policies compared to a narrative which included a woman who came from low socio-economic status. Further research needs to be conducted that will help to disentangle the role of SES and race in public perceptions of substance use and harm reduction [66]. As well, seeking the opinions of journalists who have covered the overdose crisis as well as the newspaper editors who often decide on the title of the article/text would be helpful in determining how news media organizations decide who to interview and how they frame harm reduction messaging.

To summarize, despite evidence from our content analysis indicating that bereaved mother's harm reduction messaging is being conveyed in ways that are supportive of harm reduction, our thematic analysis of newspaper coverage indicated that these advocacy messages were often obscured by reporting that emphasized a mother's grief, the biographical stories of the deceased child over structural determinants of drug-related harms and rhetorical language that continued to stigmatize and create division between different groups of people who use drugs. These findings lend themselves to a number of practical implications for both members of the news media industry as well as family advocates themselves. Stigmatizing language towards PWUD was frequently observed in the content analysis of newspaper texts included in this study. People who use drugs have themselves commented that ". . . media coverage can

be sensationalistic, exaggerating the harms of drugs and contributing to misinformation and damaging stereotypes" [67, p.198]. While the *Associated Press (AP) Stylebook* updated recommendations in 2017 to ask that stigmatizing terms to describe substance use disorder be avoided [68], evidence suggests this change has had limited impact [69]. In Canada for example, the Canadian Broadcast Corporation (CBC) continues to use the term 'addict' despite calls to eliminate its use in their reporting[45]. There is a need for journalists and family advocates to consider the use of person-first language that is non stigmatizing as well as consult best practice guidelines when speaking about drug use and PWUD in the press [70]. While not viewed as formally recognized Canadian standards on substance use and harm reduction in news media reporting, one document that offers some guidance when reporting on mental health and addiction has been published by the Canadian Journalism Forum on Violence and Trauma [71]. Another suggestion put forth has been the consideration of contact-based training sessions for members of the news media to enhance meaningful interactions and understandings of the experiences of PWUD [20].

Actively seeking out and including the voices of PWUD (who undoubtedly face multiple barriers in sharing their story) by members of the press and family members creates an opportunity for enhancing the stories being shared by bereaved mothers as well as emphasizing the complexities that are inherent in drug policy reform. Examples of advocacy groups active across Canada that represent the needs of people who use drugs include Canadian Association of People Who Use Drugs (CAPUD), the Alberta Alliance Who Educate and Advocate Responsibly (AAWEAR) and Vancouver Area Network of Drug Users (VANDU). Mother's accounts of substance use and the overdose crisis will not always align with the accounts of PWUD and in some cases, PWUD may not agree with their loved ones' perspectives on substance use. Including the perspective of those closest to the issue of drug use (those using drugs) alongside the family perspective as shared by bereaved mothers remains critical to achieving policy and practice changes that are meaningful and long lasting. Last, our content analysis showed that practical supports on the topic of substance use were rarely mentioned in this population of newspaper texts (15.9%). This represents a missed opportunity for media coverage to provide practical assistance to the public about substance use harms and ways to mitigate those harms.

Parent advocates themselves may want to reflect on the findings of this study and consider inviting PWUD to participate in interviews with them as well as consult relevant resources that offer specific strategies on how to interact with the news media more effectively [72, 73]. In light of our findings, both bereaved mothers and journalists may also want to increasingly consider mentioning the names of those individuals responsible for overseeing government responses to drug-related harms and deaths in the future. In doing so, the public's attention has the potential to be re-directed away from the individual stories of those who have passed away and towards those who have the power to change the structures that continue to allow drug-related harms to exist in our communities. As well, highlighting the impact of poverty, racism/colonialism, housing insecurity and barriers to health services alongside stories of personal struggle, trauma, grief and loss by both family advocates and journalists has the potential to amplify public understanding of the need for harm reduction services and broader systems change.

We acknowledge a number of limitations to this study. First, the small number of included texts precluded us from statistically examining whether there were associations between time period or geographical area and selected content characteristics. Second, as our included texts were limited to those pertaining to harm reduction, we suspect that the overall number of articles featuring a bereaved mother would be far greater had we included any articles related to the overdose crisis in general, or conducted additional data collection to include texts

published after 2016. This limitation is particularly important to note as bereaved mother's media representations in Canada began to expand considerably in 2016. Moms Stop the Harm for example, has increased its membership from its three original founding members in 2016 to nearly 4000 members across Canada. As a result, the volume of newspaper coverage featuring bereaved mothers since that time as well as general discourses captured in today's newspaper articles might differ substantially than in years past. It is also important to note that numerous substance use initiatives and policies have emerged in Canada since 2016 with some examples being expanded opioid-agonist treatment (including injectable opioid agonist treatment), safer supply programs, drug checking services, use of overdose phone apps, and a three-year pilot to evaluate decriminalization of a small amount of specific illegal drugs in the province of British Columbia. None-the-less, texts from our selected time period before 2016 are of historical interest as this was when many harm reduction initiatives were first being adopted in Canada. As well, the current dataset allows for future comparisons to be made between different time periods in the assessment of bereaved mothers media messaging and advocacy work. Third, we were also unable to confirm what the motivations and messaging intent of bereaved mothers was, having only the opportunity here to analyze how newspaper coverage of bereaved mothers appeared. While outside the scope of the present study, it is possible that there are differences amongst bereaved mothers within advocacy organizations in how harm reduction is defined, whether such initiatives are important to addressing overdose harms and death and if supported, how this should be conveyed to the media. It is unknown for example, how bereaved mother-advocates would interpret our finding that the media used rhetorical devices to differentiate child deaths from affluent supportive families vs. street-involved people who use drugs. Similarly, it would be important to document in future research the extent to which bereaved mother advocates agree or disagree with media positioning that downplays the idea that drug harms are structurally and socially produced. Fourth, two of our three news databases did not consistently link to the original newspaper PDFs which made it impossible to retrieve all of the photographs associated with these texts. Future research which includes analysis of photographs or televised news could provide additional insight into the underlying semiotics and discourses present in Canadian newspaper coverage on harm reduction which feature a bereaved mother whose child's death was related to substance use. Fifth, Canadian French language newspapers were not included in the retrieval of our data and texts featuring bereaved mothers were limited to the ten provinces as bereaved mothers' texts were not found in newspapers from the three Canadian territories. Finally, while it is widely recognized that 'print only' newspaper readership has decreased [74], others have argued that newspapers are still involved in 'breaking' significant stories and are often integrated into television and radio programs [75]. Our analysis did not include television, radio or social media. All of these media forms need to be considered for future drug policy media research alongside studies which investigate whether harm reduction is described differently by conservative vs. liberal news media outlets.

Conclusion

As public dialogue on harm reduction evolves in the Canadian news media, bereaved mothers will continue to represent a unique and important voice in calls for drug policy reform. Our review of Canadian newspaper texts (2000–2016) indicated that 1.1% of articles featured a mother whose child's death was related to substance use. While bereaved mothers' pro-harm reduction messages appeared in the majority of texts we examined, the news media also emphasized mothers' expressions of grief, obscured broader structural factors contributing to substance use harm and created rhetorical divisions between different groups of people who

use drugs. Such occurrences represent a bona fide threat to the media advocacy undertaken by bereaved mothers advocating for harm reduction in Canada. While recognizing the challenging circumstances that journalists currently work under [72], particularly when covering stories that are trauma-related [76], today's news outlets have an important opportunity to more accurately represent the voices of bereaved mothers and people who use drugs in ways that align more closely with their advocacy messages in support of harm reduction initiatives. In doing so, the Canadian news media has the potential to shift public opinion and government support in favour of these life-saving services.

Supporting information

S1 Table. Newspapers with highest circulation in each province/territory, Canadian harm reduction policy project (CHARPP).
(PDF)

S2 Table. Database-specific controlled vocabularies for harm reduction.
(PDF)

Author Contributions

Conceptualization: Heather Morris R. N., T. Cameron Wild, Rebecca Haines-Saah, Petra Schulz, Diane Kunyk, Tania Bubela, Elaine Hyshka.

Data curation: Heather Morris R. N., T. Cameron Wild, Marina Giovannoni, Jakob Koziel, Elaine Hyshka.

Formal analysis: Heather Morris R. N., T. Cameron Wild, Marina Giovannoni, Rebecca Haines-Saah, Jakob Koziel, Hauwa Bwala, Elaine Hyshka.

Funding acquisition: T. Cameron Wild, Elaine Hyshka.

Investigation: Heather Morris R. N., T. Cameron Wild, Elaine Hyshka.

Methodology: Heather Morris R. N., T. Cameron Wild, Marina Giovannoni, Rebecca Haines-Saah, Jakob Koziel, Petra Schulz, Diane Kunyk, Tania Bubela, Elaine Hyshka.

Project administration: Heather Morris R. N., T. Cameron Wild, Marina Giovannoni, Elaine Hyshka.

Resources: Heather Morris R. N., T. Cameron Wild, Elaine Hyshka.

Software: Heather Morris R. N., T. Cameron Wild, Jakob Koziel, Hauwa Bwala, Elaine Hyshka.

Supervision: T. Cameron Wild, Rebecca Haines-Saah, Diane Kunyk, Tania Bubela, Elaine Hyshka.

Validation: T. Cameron Wild, Elaine Hyshka.

Visualization: Heather Morris R. N., T. Cameron Wild, Elaine Hyshka.

Writing – original draft: Heather Morris R. N., T. Cameron Wild, Elaine Hyshka.

Writing – review & editing: Heather Morris R. N., T. Cameron Wild, Marina Giovannoni, Rebecca Haines-Saah, Jakob Koziel, Petra Schulz, Hauwa Bwala, Diane Kunyk, Tania Bubela, Elaine Hyshka.

References

1. Ahmad F.B., Cisewski J.A., Rossen L.M. & Sutton P. National Center for Health Statistics. Provisional drug overdose death counts [Internet]. Hyattsville, Maryland; 2023 [cited 2023 Apr 16]. Available from https://www.cdc.gov/nchs/nvss/vsrr/drug-overdose-data.htm
2. Public Health Agency of Canada, Special Advisory Committee on the Epidemic of Opioid Overdoses. Opioid- and stimulant-related harms in Canada [Internet]. Ottawa: Public Health Agency of Canada; 2023 [cited 2023 April 16]. Available from: https://health-infobase.canada.ca/substance-related-harms/opioids-stimulants/
3. Moms Stop the Harm [Internet]. 2023. Vancouver: Moms Stop the Harm. Available from: https://www.momsstoptheharm.com/
4. Streetworks. Harm Reduction Definition. 2015; Edmonton, AB: Streetworks.
5. Harm Reduction International. What is harm reduction? [Internet]. London, U.K: Harm Reduction International; Harm Reduction International; 2022. [Accessed 2023 Apr. 14]. Available from https://www.hri.global/what-is-harm-reduction
6. Irvine M.A., Kuo M., Buxton J., Balshaw R., Otterstatter M., Macdougall L., Milloy M.J., Bharmal A., Henry B., Tyndall M., Coombs D. & Gilberta M. Modelling the combined impact of interventions in averting deaths during a synthetic-opioid overdose epidemic. Addiction. 2019; 114 (9): 1602–1613. https://onlinelibrary.wiley.com/doi/abs/10.1111/add.14664 PMID: 31166621
7. Potier C., Laprevote V., Dubois-Arber, Cottencin O., & Rolland B. Supervised injection services: what has been demonstrated? A systematic literature review. Drug Alcohol Depend. 2014; 145: 48–68. https://doi.org/10.1016/j.drugalcdep.2014.10.012 PMID: 25456324
8. Kennedy M.C., Karamouzian M. & Kerr T. Public health and public order outcomes associated with supervised drug consumption facilities: A systematic review. Curr. HIV/AIDS Rep. 2017; 14(5), 161–183. https://doi.org/10.1007/s11904-017-0363-y PMID: 28875422
9. Kennedy M.C., Hayashi K., Milloy M-J, Wood E. & Kerr T. Supervised injection facility use and all-cause mortality among people who inject drugs in Vancouver, Canada: A cohort study. PloS Med. 2019; 16 (11): 1–20. https://journals.plos.org/plosmedicine/article?id=10.1371/journal.pmed.1002964 PMID: 31770391
10. Wild C.T., Koziel J., Anderson-Baron J., Asbridge M., Belle-Isle L., Dell C., Elliott R., Hathaway A., MacPherson D., McBride K., Pauly B., Strike C., Galovan A. & Hyshka E. Public support for harm reduction: A population survey of Canadian adults. *PloS one*. 2021; 16(5): 1–18. https://journals.plos.org/plosone/article?id=10.1371/journal.pone.0251860 PMID: 34010338
11. Entman R.M. Framing: Toward clarification of a fractured paradigm. J. Commun. 1993; 43(4): 51–58. https://doi.org/10.1111/j.1460-2466.1993.tb01304.x
12. Lancaster K., Hughes C., Spicer B., Mathew-Simmons F. & Dillon P. Illicit drugs and the media: Models of media effects for use in drug policy research. Drug Alcohol Rev. 2011; 30(4): 397–402. https://doi.org/10.1111/j.1465-3362.2010.00239.x PMID: 21355898
13. McGinty E., Kennedy-Hendricks A., Baller J., Niederdeppe J., Gollust S. & Barry C. Criminal activity or treatable health condition? News media framing of opioid analgesic abuse in the United States, 1998–2012. Psychiatr Serv. 2016; 67(4): 405–411. https://doi.org/10.1176/appi.ps.201500065 PMID: 26620290
14. Kennedy L. & Valleriani J. "Everybody loves a redemption story around election time": Rob Ford and media construction of substance misuse and recovery. Can. J. Criminol. Crim. Justice. 2017; 59(4): 461–497. https://doi.org/10.3138/cjccj.2017.0007
15. Kennedy-Hendricks A., Levin J., Stone E., McGinty E.E., Gollust S.E. & Barry C.L. News media reporting on medication treatment for opioid use disorder amid the opioid epidemic. Health Aff. 2019; 38(4): 643–651. https://doi.org/10.1377/hlthaff.2018.05075 PMID: 30933576
16. Webster F., Rice K. & Sud A. A critical content analysis of media reporting on opioids: The social construction of an epidemic. Soc. Sci. Med. 2020; 244, 1–9. https://doi.org/10.1016/j.socscimed.2019.112642 PMID: 31731136
17. Wild C.T., Koziel J., Anderson-Baron J., Hathaway J., McCurdy A., Xu X, Asbridge M., Belle-Isle L., Hathaway A., MacPherson D. & Hyshka E. Media coverage of harm reduction, 2000–2016: A content analysis of tone, topics, and interventions in Canadian print news. Drug Alcohol Depend. 2019; 27 (205), 1–9. https://doi.org/10.1016/j.drugalcdep.2019.107599 PMID: 31610295
18. McGinty E.E., Stone E.M., Kennedy-Hendricks A., Sanders K., Beacham A., & Barry C. U.S. news media coverage of solutions to the opioid crisis, 2013–2017. Prev. Med. 2019-a; 126: 1–6. https://doi.org/10.1016/j.ypmed.2019.105771

19. Quan A.M.L., Wilson L.A., Mithani S.S., Zhu D.T., Bota A.B. & Wilson K. Reporting on the opioid crisis (2000–2018): Role of *The Globe and Mail*, a Canadian English-language newspaper in influencing public opinion. Harm Reduct. J. 2020; 17(93): 1–11. https://doi.org/10.1186/s12954-020-00443-7

20. McGinty E.E., Stone E.M., Kennedy-Hendricks A. & Barry C. Stigmatizing language in news media coverage of the opioid epidemic: Implications for public health. Prev. Med. (2019-b); 124: 110–114. https://doi.org/10.1016/j.ypmed.2019.03.018 PMID: 31122614

21. Kelly J. F. & Westerhoff C.M. Does it matter how we refer to individuals with substance-related conditions? A randomized study of two commonly used terms. Int. J. Drug Policy. 2010; 21(3), 202–207. https://doi.org/10.1016/j.drugpo.2009.10.010 PMID: 20005692

22. Socia K.M., Stone R., Palacios W.R. & Cluverius J. Focus on prevention: The public is more supportive of 'overdose prevention sites' than they are of 'safe injection facilities'. Criminol. 2021; 20: 729–754. https://onlinelibrary.wiley.com/doi/full/10.1111/1745-9133.12566

23. Benford R.D. & Snow D.A. Framing processes and social movements: An overview and assessment. Annu. Rev. Sociol. 2000; 26: 611–639. https://doi.org/10.1146/annurev.soc.26.1.611

24. Snow D.A. Framing and social movements. In: Snow, editor, Wiley Online Encyclopedia of Social and Political Movements. Hoboken, NJ: John Wiley & Sons; 2013. https://doi.org/10.1002/9780470674871.wbespm434

25. Insights GG. What is social injustice? Effective strategies for change [Internet]. Las Vegas; 2023 June 30 [Accessed 2023 Sept 25]. Available at https://www.graygroupintl.com/blog/what-is-social-injustice

26. Wallack L. & Dorfman L. Media advocacy: A strategy for advancing policy and promoting health. Health Educ. Q. 1996; 23(3): 293–317. https://doi.org/10.1177/109019819602300303 PMID: 8841816

27. Jernigan D.H. & Wright P.A. Media advocacy: Lessons from community experiences. J. Public Health Policy. 1996; 17(3): 306–330. https://doi.org/10.2307/3343268 PMID: 8918021

28. Dorfman L. & Daffner Krasnow I. Public health and media advocacy. Annu. Rev. Public Health. 2014; 35: 293–306. https://doi.org/10.1146/annurev-publhealth-032013-182503 PMID: 24328989

29. Clark C.D. How sensationalism compounds the opioid crisis. The Washington Post. 2017 July 5 [Cited 2023 Apr. 14]. Available from https://www.washingtonpost.com/news/made-by-history/wp/2017/07/05/how-sensationalism-compounds-the-opioid-crisis/

30. Morris H., Schulz P., Jenkins E., Haines-Saah R. & Hyshka E. 'I'm not your reality show': Perspectives of bereaved mothers' engagement with the news media to advance drug policy reform. Soc. Sci Med. Qualitative Res in Health. 2021; 1: 1–8. https://doi.org/10.1016/j.ssmqr.2021.100011

31. Bachhuber M.A., McGinty E.E., Kennedy-Hendricks A., Niederdeppe J., Barry C.L. Messaging to increase public support for naloxone distribution policies in the United States: Results from a randomized survey experiment. *PLOS one*. 2015; 10(7): http://journals.plos.org/plosone/article?id=10.1371/journal.pone.0130050 PMID: 26132859

32. Sumnall H.R., Atkinson A.M., Trayner K.M.A., Gage S.H. & McAuley A. (2020). Effects of messaging on public support for drug consumption rooms in Scotland, UK. Int. J. Drug Policy. 2020; 83: 1–10. https://doi.org/10.1016/j.drugpo.2020.102855 PMID: 32693322

33. Creswell J.W. & Creswell J.D. Research design: Qualitative, Quantitative, and Mixed Methods Approaches (5th Ed.). Los Angeles: Sage; 2018.

34. Neuendorf K.A. The content analysis guidebook. 2nd ed. Los Angeles: Sage; 2017.

35. Atkinson A.M., McAuley A., Trayner K.M.A. & Sumnall H.R. 'We are still obsessed by this idea of abstinence': A critical analysis of UK news media representations of proposals to introduce drug consumption rooms in Glasgow, UK. Int. J. Drug Policy. 2019; 68: 62–74. https://doi.org/10.1016/j.drugpo.2019.03.010 PMID: 31003193

36. Krippendorff K. Content analysis: An introduction to its methodology. 4th ed. Los Angeles: Sage; 2019.

37. Braun V. & Clarke V. Using thematic analysis in psychology. Qual Psychol. 2006; 26(3); 417–426. https://doi.org/10.1191/1478088706qp063oa

38. Braun V, Clarke V, Terry G. Thematic analysis. In: Rohleder P & Lyons AC, editors. Qualitative Research in Clinical and Health Psychology. Basingstoke: Palgrave Macmillan; 2015. p. 95–113.

39. Maguire M. & Delahunt B. Doing a thematic analysis: A practical, step-by-step guide for learning and teaching scholars. AISHE-J 2017; 8(3): 3351–3351. https://ojs.aishe.org/index.php/aishe-j/article/view/335

40. Lincoln Y.S. & Guba E.G. Naturalistic inquiry. Newbury Park, CA: Sage; 1985.

41. Government of Canada. Changing how we talk about substance use* [Internet]. Ottawa: Government of Canada. 2013 [cited 2023 Apr 14]. Available at https://www.canada.ca/content/dam/hc-sc/documents/services/publications/healthy-living/problematic-substance-use/substance-use-eng.pdf

42. Shatterproof. Addiction Language Guide. Shatterproof; 2021 Feb 26 [Accessed 2023 Apr 14]. Available from https://www.shatterproof.org/our-work/ending-addiction-stigma/change-your-language

43. Ogden J. Want to hook your readers? Apply these 10 principles to create captivating news stories [Internet]. 2014 Oct. 15 [Accessed 2023 Apr 14]. Available from https://copyblogger.com/captivating-news-stories/

44. Crear-Perry J., Correa-de-Araujo R., Lewis Johnson T., McLemore M.R., Neilson E.& Wallace M. Women's Health 2021; 30(2),. Social and structural determinants of health inequities in maternal health. J. Women's Health, Vol. 30(2): 230–235. https://www.liebertpub.com/doi/epdf/10.1089/jwh.2020.8882

45. British Columbia Centre for Substance Use (BCCSU). Letter to Ombudsman, Canadian Broadcasting Corporation [Internet]. Vancouver; 2017 Dec. 5 [Accessed 2023 Apr 14]. Available at https://www.bccsu.ca/wp-content/uploads/2017/12/Letter-Stigmatizing-Language-in-the-Media.pdf

46. Short D. Victims of opioid crisis memorialized in online campaign: Moms Stop the Harm. Edmonton Journal. 2021 May 2, 2021[Cited 2023 Apr 14]. Available from https://edmontonjournal.com/news/local-news/victims-of-opioid-crisis-memorialized-in-online-campaign-moms-stop-the-harm

47. Rohlinger D. & Vaccaro C. Media and social movements. In: Snow, editor. Encyclopedia of social and political movements, Wiley Online Library (1–6). Hoboken, NJ: John Wiley & Sons; 2013. https://doi.org/10.1002/9780470674871.wbespm128

48. Andrews K.T. & Caren N. Making the news: Movement organizations, media attention, and the public agenda. Am. Sociol. Rev. 2010; 75(6): 841–866. https://doi.org/10.1177/0003122410386689

49. Rohlinger D.A., Kail B., Taylor M. & Conn S. Outside the mainstream: Social movement organization media coverage in mainstream and partisan news outlets. Res. Soc. Mov. Confl. Change. 2012; 33: 51–80. https://doi.org/10.1108/S0163-786X(2012)0000033006

50. Elliott T.A., Amenta E. & Caren N. Recipes for attention: Policy reforms, crises, organizational characteristics, and newspaper coverage of the LGBT movement, 1969–2009. Sociol. Forum. 2016; 31(4): 926–947. https://doi.org/10.1111/socf.12290

51. Rohlinger D.A. & Brown J. Mass media and institutional change: Organizational reputation, strategy, and outcomes in the academic freedom movement. Mobilization. 2013; 18(1): 41–64. https://doi.org/10.17813/maiq.18.1.q6h62418323x7858

52. Canadian Public Health Association. Public health: A conceptual framework (2nd Ed.) [Internet]. Ottawa; March 2017 [Accessed 2023 Sept 25]. Available at https://www.cpha.ca/public-health-conceptual-framework

53. Government of Canada. Consultation on the prescription drug list: Naloxone [Internet]. Ottawa: Government of Canada. 2016 [cited 2023 Apr. 14]. Available at https://www.canada.ca/en/health-canada/services/drugs-health-products/public-involvement-consultations/drug-products/consultation-prescription-drug-list-naloxone.html

54. Hart C. Drug use for grown-ups: Chasing liberty in the land of fear. New York: Penguin Books; 2021.

55. Fine G. A. Public narration and group culture: Discerning discourse in social movements. In: Johnston H. & Klandermans B., editors. Social movements, protest and contention, Volume 4. Minneapolis: University of Minnesota Press; 2003, pp. 127–143.

56. Iyengar S. Is anyone responsible? How television frames political issues. Chicago, IL: University Press; 1991

57. Kennedy-Hendricks A., McGinty E.E. & Barry C. Effects of competing narratives on public perceptions of opioid pain reliever addiction during pregnancy. J Health Polit Policy Law. 2016; 41(5): 873–916. https://doi.org/10.1215/03616878-3632230 PMID: 27256811

58. First Nations Health Authority. Overdose data and First Nations in BC: Preliminary findings [Internet]. West Vancouver, BC: First Nations Health; 2017 [Accessed 2023 Apr 16]. Available from https://www.fnha.ca/AboutSite/NewsAndEventsSite/NewsSite/Documents/FNHA_OverdoseDataAndFirstNationsInBC_PreliminaryFindings_FinalWeb_July2017.pdf

59. Government of Alberta. Opioids and substances of misuse among First Nations People in Alberta: Alberta Report [Internet]. Edmonton: Government of Alberta. 2017 [cited 2023 Apr 14]. Available from https://open.alberta.ca/dataset/cb00bdd1-5d55-485a-9953-724832f373c3/resource/31c4f309-26d4-46cf-b8b2-3a990510077c/download/opioids-substances-misuse-report-firstnations-2017.pdf

60. The Alberta First Nations Information Governance Centre and Government of Alberta. Alberta Opioid Response Surveillance Report: First Nations People in Alberta [Internet]. Edmonton: Government of Alberta; 2021 June. [Accessed 2023 Apr. 16]. Available from \https://open.alberta.ca/dataset/ef2d3579-499d-4fac-8cc5-94da088e3b73/resource/1d3c4477-7a5b-40a8-90f0-a802dbfd7748/download/health-alberta-opioid-response-surveillance-report-first-nations-people-2021-06.pdf

61. James K. & Jordan A. (2018). The opioid crisis in Black communities. J Law Med Ethics. 2018; 46: 404–421. https://doi.org/10.1177/1073110518782949 PMID: 30146996

62. Johnston G. The kids are all white: Examining race and representation in news media coverage of opioid overdose deaths in Canada. Sociol Inq. 2019; 90(1): 1–24. https://onlinelibrary.wiley.com/doi/abs/10.1111/soin.12269

63. McLean K. From "junkies" to "soccer moms": Newspaper representations of overdose, 1988–2014. Crit. Criminol. 2017; 25: 411–432. https://doi.org/10.1007/s10612-017-9350-2

64. Mendoza S., Rivera A.S. & Hansen H.B. Re-racialization of addiction and the redistribution of blame in the white opioid epidemic. Med. Anthropol. Q. 2018; 33(2): 242–262. https://doi.org/10.1111/maq.12449 PMID: 29700845

65. Netherland J. & Hansen H.B. (2016). The war on drugs that wasn't: Wasted whiteness, "dirty doctors," and race in media coverage of prescription opioid misuse. Cult Med Psychiat. 2016; 40: 664–686. https://doi.org/10.1007/s11013-016-9496-5 PMID: 27272904

66. McGinty E., Pescosolido B., Kennedy-Hendricks A. & Barry C. Communication strategies to counter stigma and improve mental health and substance use disorder policy. Psychiatr Serv. 2018; 69(2), 136–146. https://doi.org/10.1176/appi.ps.201700076

67. Fraser S., Pienaar K., Dilkes-Frayne E., Moore D., Kokanovic R., Treloar C. & Dunlop A. Addiction stigma and the biopolitics of liberal modernity: A qualitative analysis. Int. J. Drug Policy. 2017; 44: 192–201. https://doi.org/10.1016/j.drugpo.2017.02.005 PMID: 28366599

68. Szalavitz M. The AP learns to talk about addiction. Will other media follow? [Internet]. Undark; 2017 [Accessed 2023 Apr. 14]. https://undark.org/2017/06/06/associated-press-stylebook-addiction/

69. Bessette L.G., Hauc S.C., Danckers H., Atayde A. & Saitz R. The associated press stylebook changes and the use of addiction-related stigmatizing terms in news media. Subst Abus. 2022; 43(1): 127–130. https://doi.org/10.1080/08897077.2020.1748167 PMID: 32348190

70. The Action Lab: Changing the Narrative Initiative. Overdose reporting style guide [Internet]. Boston; 2020 [Accessed 2023 Apr 14]. Available at https://56c8fd7b-27cc-4821-ac86-9603ad2562da.filesusr.com/ugd/dc612a_6b8f937d2b024b88b090e0ea22f423e5.pdf

71. The Canadian Journalism Forum on Violence and Trauma. Mindset: Reporting on mental health (3rd Ed.) [Internet]. 2020 [Accessed 2023 Sept 25]. Available at https://www.mindset-mediaguide.ca/

72. Canadian Drug Policy Coalition. Getting to tomorrow media guide: A resource for advocacy and community organizations on how to speak with and engage media. [Internet]. Vancouver; 2021 Mar. 5 [Accessed 2023 Apr 14]. Available at https://gettingtotomorrow.ca/2021/02/25/getting-to-tomorrow-media-guide/

73. Moms Stop the Harm. Moms Stop the Harm Advocates Handbook (2nd Ed.). 2017; Edmonton, AB: Author.

74. Rody B. Newspaper reach, influence continues to drop: The bright spot is community news, where reach is still strong among all demos. Media in Canada [Internet]. Brunico Communications Inc; 2020 [Accessed 2023 Apr 14]. https://mediaincanada.com/2020/10/26/newspaper-reach-influence-continues-to-drop/

75. Hayes M., Ross I.E., Gasher M., Gutsetein D., Dunn J.R. & Hackett R.A. Telling stories: News media, health literacy and public policy in Canada. Soc. Sci. Med. 2007; 64(9): 1842–1852. https://doi.org/10.1016/j.socscimed.2007.01.015 PMID: 17337317

76. CBC Radio. "An enormous change in the news agenda": How trauma-informed reporting is transforming journalism. CBC Radio. 2019 Aug 23 [Cited 2023 Apr 14]. Available from https://www.cbc.ca/radio/sunday/the-sunday-edition-for-august-25-2019-1.5256740/an-enormous-change-in-the-news-agenda-how-trauma-informed-reporting-is-transforming-journalism-1.5256757
